# Urban Growth Modeling Using Cellular Automata with Multi-Temporal Remote Sensing Images Calibrated by the Artificial Bee Colony Optimization Algorithm

**DOI:** 10.3390/s16122122

**Published:** 2016-12-14

**Authors:** Fereydoun Naghibi, Mahmoud Reza Delavar, Bryan Pijanowski

**Affiliations:** 1 GIS Department, School of Surveying and Geospatial Engineering, College of Engineering, University of Tehran, Tehran 1439951154, Iran; f.naghibi@ut.ac.ir; 2 Center of Excellence in Geomatic Engineering in Disaster Management, School of Surveying and Geospatial Engineering, College of Engineering, University of Tehran, Tehran 1439951154, Iran; 3 Department of Forestry and Natural Resources, Purdue University, 195 Marsteller Street, West Lafayette, IN 47907, USA; bpijanow@purdue.edu

**Keywords:** urban growth model, cellular automata, model calibration, swarm intelligence, artificial bee colony algorithm, remote sensing image

## Abstract

Cellular Automata (CA) is one of the most common techniques used to simulate the urbanization process. CA-based urban models use transition rules to deliver spatial patterns of urban growth and urban dynamics over time. Determining the optimum transition rules of the CA is a critical step because of the heterogeneity and nonlinearities existing among urban growth driving forces. Recently, new CA models integrated with optimization methods based on swarm intelligence algorithms were proposed to overcome this drawback. The Artificial Bee Colony (ABC) algorithm is an advanced meta-heuristic swarm intelligence-based algorithm. Here, we propose a novel CA-based urban change model that uses the ABC algorithm to extract optimum transition rules. We applied the proposed ABC-CA model to simulate future urban growth in Urmia (Iran) with multi-temporal Landsat images from 1997, 2006 and 2015. Validation of the simulation results was made through statistical methods such as overall accuracy, the figure of merit and total operating characteristics (TOC). Additionally, we calibrated the CA model by ant colony optimization (ACO) to assess the performance of our proposed model versus similar swarm intelligence algorithm methods. We showed that the overall accuracy and the figure of merit of the ABC-CA model are 90.1% and 51.7%, which are 2.9% and 8.8% higher than those of the ACO-CA model, respectively. Moreover, the allocation disagreement of the simulation results for the ABC-CA model is 9.9%, which is 2.9% less than that of the ACO-CA model. Finally, the ABC-CA model also outperforms the ACO-CA model with fewer quantity and allocation errors and slightly more hits.

## 1. Introduction

In recent decades, rapid urbanization has led to many negative impacts on the environment, such as the loss and fragmentation agricultural lands and of natural areas that support wildlife. To avoid these impacts, anticipatory planning has to be considered [1]. Urban growth models have been proposed to use the capabilities of a new generation of spatial analysis tools within the geospatial information systems (GIS) framework. They investigate urban regions using various multi-temporal datasets such as remote sensing images to detect changes over the time [2,3,4,5,6,7]. A number of models have been created in order to support land use planning [8,9,10,11,12,13] or to quantify the environmental consequences of potential future urban growth scenarios (e.g., [14,15]).

It is well accepted that urban growth is a complex process [16,17,18] consisting of several interacting elements. Thus, to model this process several urban growth models have been presented such as Markov chain models [19], spatial logistic regression [20], multi-criteria evaluation [19], cellular automata (CA) [16,21,22,23], agent-based models [17,24] and machine learning and artificial intelligence (AI) methods like artificial neural networks (ANN) [25,26], support-vector machine (SVM) [27,28], genetic algorithms (GA) [29], and data mining [30]. AI-based methods can capture existing nonlinearities and heterogeneities of the urban growth process. It is clear that superiority of methods depends on how to set the configuration parameters of the algorithms, size of training and test sets, designing classifiers in two classes or multiclass, choice of datasets for training and validation and so on [31,32].

Among the models listed above, CA has arguably been the most popular tool used to simulate urban changes because of its capacity to reproduce the dynamics of complex systems, its self-organization characteristics, and its flexibility and compatibility with the raster data structure (in sensu) [11,33,34]. Indeed, it has been shown that CA models, especially GIS-based CA models, can efficiently simulate urban growth processes [16,34,35,36] and estimate spatial patterns of urban growth [36]. However, calibrating an urban CA is challenging and complicated because of the existing spatial heterogeneity of urban forms and the nonlinearity of urban growth driving forces (cf. [37,38]). To calibrate urban CA models, some researchers (e.g., [21,30,35,39]) have used statistical approaches, such as multi-criteria evaluation (MCE) or logistic regression (LR), to estimate growth parameters, with moderate success. Fuzzy logic approaches have also been employed to estimate CA parameters; some have even used fuzzy logic to quantify uncertainties in CA models [3,40,41]. However, many researchers are still exploring how other techniques might further improve the calibration of CA-based urban models. One common approach has been to integrate CA models with other AI tools—so called “hybrid AI modeling environments”—where tools such as ANN [42,43,44], SVM [45,46], simulated annealing [47], data mining algorithms [48], and genetic algorithms [49,50,51,52] are used to calibrate CA-based urban models.

Each of the abovementioned CA calibration approaches has strengths and weaknesses. Statistical-based methods, such as MCE, assume linear relationships among spatial variables, which can neither capture nor eliminate autocorrelation effects because of non-linear relations in urban growth driving forces [47,53]. Integrating CA with ANN has some benefits as ANNs can quantify non-linear relationships among spatial variables; however, ANNs due to their somewhat “black box nature”, do not provide clear interpretable weights for different variables [41], can get trapped in local minima [42] or may even over-fit the data and thus cannot be generalized [25]. Other intelligent techniques for calibrating CA have been proposed, based on evolutionary algorithms such as genetic algorithms (GAs). Despite the advantages of GAs such as promising behavior in searching complex and multimodal spaces, GAs sometimes are computationally intense when solving small scale problems. They may not reach the global solution and suffer from being trapped in local optima [54,55].

An active area of AI research is called swarm intelligence (SI) [56,57] and this has been promising in a variety of fields such as business, medicine and information sciences. Swarm intelligence is the simulation of social insects using computer models. The simulation takes advantage of the simple behavior of individuals (e.g., ants following each other in a line) but together their “meta” behavior (e.g., searching for food) emerges as a complex outcome that is robust, flexible and efficient. Swarm behaviors that are simulated include ant colony movement, bird flocking, and fish schooling, among others [58]. Several SI algorithms exist, some of which researchers in urban modeling have employed to a very limited extent. Liu et al. used an ant colony optimization (ACO) approach to discover CA transition rules [59,60]. Their ACO-CA model effectively captured the complexities of urban growth. More recently, Yang et al. (2013) showed that integrating CA with a bee colony optimization (BCO) algorithm could produce better simulation results than an ACO-CA model [61]. Feng et al. tested a particle swarm optimization (PSO)-CA model with a model developed using LR and found that PSO increased model goodness of fit in four out of five criteria over using LR [62]. Cao et al. used the cuckoo search algorithm to discover transition rules for CA. They showed that the CA model calibrated by that cuckoo search algorithm had greater accuracy than that of other swarm models such as the ACO-CA model [63].

The artificial bee colony (ABC) algorithm was developed over ten years ago and is one of the SI algorithms most widely used by businesses to understand complex human social systems. ABC simulates the foraging behavior of honey bee colonies [64]. The ABC has shown better results in performance and computational efficiency compared to other SI algorithms like PSO and ACO [65,66]. ABC has several advantages over these other SI approaches as ABC algorithms display simplicity, flexibility, and robustness; fewer control parameters are also required compared to alternative methods and there is considerable ease of implementation as well [67].

Here, we present our new approach that integrates ABC and a LR-based CA model for urban growth simulation. Our research attempted to continue the way to explore new effective methods. To the best of our knowledge, integration of ABC with CA for the purposes of characterizing transition rules (CA calibration) has not been attempted. Similar research has been carried out, however. Yang et al. recently integrated a BCO algorithm with CA. BCO, like ABC, is inspired by a bee’s food searching behavior [61]. However, BCO builds solutions from the beginning within execution steps while ABC performs a local search to improve a “current best solution” iteratively [68]. However, we believe that the integration of CA with SI algorithms such as ABC is at the forefront of CA and hybrid AI urban modeling research.

The proposed model was tested on Urmia (Iran) to simulate its future urban growth patterns. The city is located in the northwest of Iran and is an important geographical region bordering two countries (Turkey and Iraq). In the last five decades, the population of Urmia has increased more than 10-fold, while its area contemporarily increased by about 27-fold [69], showing rapid urbanization.

## 2. CA Model for Urban Growth

Urban growth models attempt to anticipate future urban expansion with increasing benefits and reducing environmental impacts. Some important parameters affecting urban growth include accessibility to the central business district, population centers, main roads and railways, proper physical condition (such as slope, elevation, distance to faults, water courses and land capabilities), considering zoning policies and distance to sensitive ecological areas [36]. One of the successful methods for simulating spatial dynamic urban growth is the use of CA. However, what can influence the success of CA in urban growth simulation is the optimum determination of the CA transition rules based on not only the cell status and those of its neighbors but also other factors called development potentials and constraints such as environmentally sensitive areas, restricted areas and development policies. Development potentials and constraints are, following several others [17,62]:

(1) $$S_{ij}^{t+1}=f\left(S_{ij}^{t},\Omega_{ij}^{i},Con,N\right)$$

where $S_{ij}^{t}$ and $S_{ij}^{t+1}$ show the states of a cell at location *ij* at time *t* and *t* + 1, respectively. *f* is the transition function, specifying $\Omega_{ij}^{i}$ as the neighborhood evaluation function, Con as the constraints of influencing factors, which exclude the urban growth such as sensitive and protecting areas. This can be defined as a binary layer that was prepared by overlaying the exclusion areas. In this layer, the zero value shows areas where development is not allowed, and *N* is the number of cells. Therefore, the development potential value ${\left(Pc\right)}_{ij}^{i}$ of the cell at location *ij* at time *t* is formulated as follows [45,62]:

(2) $${\left(Pc\right)}_{ij}^{t}={\left(Pg\right)}_{ij}\times \Omega_{ij}^{t}\times Con\times \left(1+{\left(-ln\gamma \right)}^{\alpha }\right)$$

where ${\left(Pg\right)}_{ij}$ is a potential value of cell at location *ij*. γ is a stochastic factor ranging from 0 to 1, α is a parameter to control the degree of stochasticity. LR is a multi-variant discovery method that is often combined with CA. LR has the capability to eliminate spatial autocorrelation and reduce spatial dependency among variables. Formally, by using LR, the development potential of cell at location *ij*, ${\left(Pg\right)}_{ij}$, based on spatial factors is presented as [37,38,62]:

(3) $${\left(Pg\right)}_{ij}=\frac{1}{1+exp\left(-\left(a_{1}x_{1}+\ldots +a_{k}x_{k}\right)\right)}$$

where $x_{1},x_{2},\ldots ,\;x_{k}$ are various spatial development variables affecting urban growth, including the aforementioned accessibility to the central business district, population centers, main roads and railways, suitable physical conditions (such as slope, elevation, distance to faults, water courses and land capabilities), considering zoning policies and distance to sensitive ecological areas [17,36]. $a_{1},a_{2},\ldots ,\;a_{k}$ are different weights for the variables. Additionally, the urban growth is influenced by dynamic factors such as the local interaction between a cell at location *ij* and its neighborhood cells. The potential of neighborhood effects ($\Omega_{ij}^{t}$) within an *n* × *n* Moore’s neighborhood of the cell at location *ij* is represented as follows [36]:

(4) $$\Omega_{ij}^{t}=\frac{\sum_{ij}Con(S_{ij}^{t}=urban)}{n\times n-1}$$

where $Con\left(S_{ij}^{t}=urban\right)$ shows the number of urban cells in Moore’s neighborhood. $S_{ij}^{t}$ is situation of cell at location *ij* that can be urban or non-urban. Finally, the value of the calculated development potential ${\left(Pc\right)}_{ij}^{t}$ is compared with a threshold value (Q) to decide whether a non- urban cell can be converted to an urban cell at time *t +* 1 using Equation (5). Q is a uniform random distribution grid in range of [0, 1] [36,39]. This threshold can be easily found out through a couple of trials [70]:

(5) $$S_{ij}^{t+1}=\{\begin{array}{ll} Converted\;to\;urban & {\left(Pc\right)}_{ij}^{t}\geq Q\\ Non\;converted & {\left(Pc\right)}_{ij}^{t}<Q \end{array}$$

## 3. The Artificial Bee Colony (ABC) Algorithm

The ABC algorithm is inspired by the intelligent behavior of honeybees in finding nectar sources around their hives. It mimics a real bee colony, where tasks are completed by specialized bees with a division of tasks distributed in three kinds of groups [64,71]. Exploitation of the explored nectar sources and sharing information about their quality are performed by the worker bees. Scout bees search the surrounding environment to discover new food sources, and onlooker bees wait in the hive to receive information about the explored food sources provided by the worker bees. Onlooker bees always select a food source with the highest quality. Communications between these different groups are often accomplished through a dance, where onlooker bees in the hive watch dances that show profitable food sources and choose a source based on the appropriateness of dances [65]. The ultimate goal of the group is to optimize a unified outcome, viz., store the greatest amount of nectar (food) in the hive.

In the optimization problem, the ABC algorithm considers the food source position and the amount of its nectar as a possible solution for the problem and fitness of the solution, respectively. The following steps represent the basic ABC algorithm procedure as described by others [64,72,73]:

In first step, it is necessary to perform exploration of the search space. This can be done by adjusting all worker bees randomly. Random initialization is accomplished via:

(6) $$X_{mn}=X_{min,n}+rand\left(0,1\right)\left(X_{max,n}-X_{min,n}\right)$$

where $X_{mn}$ is a solution (food source), *m* = 1…*SN*, *n* = 1…*D*. *SN* is the number of solutions (food sources) and *D* represents the number of optimization parameters. In each of the iterations, a worker employed bee is randomly assigned to the food source (the solution). The selected worker bee applies a modification on the position of the food source according to its local information to find a new food source. The food source $V_{mn}$ is located within the neighborhood of every food source $X_{mn}$:

(7) $$V_{mn}=V_{mn}+\varphi_{mn}\left(X_{mn}-X_{zn}\right)$$

where *n* is a random number in [1, *D*] and z∈{1,2,...SN} is a random index that must be different from *m* so that a new hybrid solution can be obtained. $\varphi_{mn}$ represents a uniformly distributed real number in range of [−1, 1] that controls production of food source locations around $X_{mn}$. In this equation, the *m*th worker bee exchanges information with the *z*th worker bee. Then, a greedy selection strategy is made to choose a better position based on the profitability between the new position ($V_{mn}$) and the previous position ($X_{mn}$). The profitability (fitness) of the two positions is evaluated by:

(8) $$fitness_{i}=\left\{\begin{array}{ll} \frac{1}{1+f_{i}} & if\;f_{i}\geq 0\\ 1+abs\left(f_{i}\right) & if\;f_{i}<0 \end{array}\right\}$$

where $f_{i}$ is the cost value of the produced solution *i* and *abs* is the absolute value. If the new position has a better fitness value, then the previous position is ignored. Otherwise, the previous position is preserved and the worker bee remains at that position. Based on following probability, every one of the onlooker bees decides to exploit the position of worker bees around its location or not:

(9) $$p_{i}=\frac{fitness_{i}}{\sum_{i=1}^{SN}fitness_{i}}$$

Again, the onlooker bees search around corresponding worker bees through Equation (7) and a greedy selection strategy is performed on the onlooker bees in the mentioned way. Then, the abandoned solution (position) is determined if it exists and is replaced with a new randomly produced solution (position) $X_{mn}$ for the scout using Equation (7). The best solution (position) achieved so far is memorized and the previous steps are repeated until reaching the termination criterion. The pseudo-code of the ABC algorithm is given in Algorithm 1:
**Algorithm 1.** The pseudo-code of the ABC algorithmGenerate the initial solution population using Equation (6) Set *Cycle* = 1 **Repeat** until *Cycle* ≤ the maximum iteration number Produce positions (new solutions or food source positions) for worker bees by Equation (7) and evaluate them Apply the greedy selection process to select worker bees Calculate the probability values *p_i_* using Equations (8) and (9) **If**
*p_i_* > *rand(0,1),*
**then**  Produce positions (the new solutions) for onlooker bees using Equation (7) and evaluate them  Apply the greedy selection process to select onlooker bees **End if** Determine the abandoned position (solution), if exists Replace it with a new randomly produced position for the scout using Equation (7) Record current best solution *Cycle* = *Cycle* + 1 **End repeat**

## 4. The New ABC-CA Model

Here, we combine the ABC algorithm with CA to determine a set of optimum transition rules for the CA portion of the urban model. In the first step, CA components such as cell size and the neighborhood size (in this study radius neighborhood was defined within three cells) were established. The input data which includes initial land-use maps, spatial variables as urban growth driving forces and urban expansion limitations are contained as raster format maps.

Then, the initial configurations of the ABC algorithm were set up. In this research, population size, number of worker bees and number of onlooker bees were set to 100, 50 and 50, respectively. The dimension in the proposed ABC-CA model is set to 7, showing the number of spatial variables and limitation conditions (distance from business center, distance from road networks, distance from population centers, land use, environmental sensitive areas, slope and elevation maps). Our proposed model was repeated 350 times.

**Figure 1:**
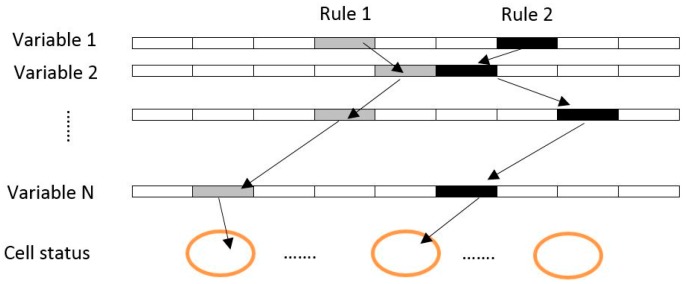
The principle of structuring CA rules based on the ABC algorithm [61].

Also, initial CA transition rules were randomly set by the worker bees. A transition rule is produced by a value of the cell status. This status can be defined by the lower and upper bound of each parameter. The cell status and variables illustrated in Figure 1 are linked to a rule with the same pattern.

According to Figure 1, every spatial variable (the urban growth driving forces) can be divided into various classes, for example variable slope can be classified into 0%–5%, 5%–7%, 7%–10%, 10%–15% and more than 15%. Each of these classes can be considered as a variable affecting urban growth. With each iteration of the ABC algorithm, and for every variable (including its subset classes), best lower and upper threshold values are determined. A transition rule is constructed by defining an upper and a lower threshold value for each variable. Worker and onlooker bees attempt to find the best lower and upper threshold values of each variable. The combination of a set of optimized values forms a transition rule based on the selection performed by the ABC algorithm. In addition, development potential maps for each defined bee are produced through LR (Equation (3)) based on the optimized values (the lower and upper threshold values) of variables. In other words, the potential or the possibility of conversion of a non-urban cell to an urban cell is calculated by LR. This process is performed in CA mechanism. In each encoded solution of the population of bees, the parameters such as Dist-road, Slope, Elevation, Dist_population_centerss and Dist-Business_center lower, and upper limitations, are calculated. A sample of transition rules of CA can be represented in Table 1: 

Finally, the CA output will be a map showing simulation of urban growth for each worker bee. By cell-based comparison among the produced simulated maps for every bee with the initial land-use map, the best bee is selected according to maximum likelihood with the initial land-use map. If the results of the simulation are recognized to be appropriate, the process is completed and these new transition rules are stored. Otherwise, based on the second step of the ABC algorithm, the better worker bee applies modifications on the solutions (rules) according to feedbacks ($V_{mn}$ in Equation (7)). Other steps of ABC algorithm also continue to search for an optimal solution (rule). The combination of the optimized coefficient values as a path obtained for each variable through the ABC algorithm constitutes our CA transition rules (for example mentioned above rule).

**Figure 2:**
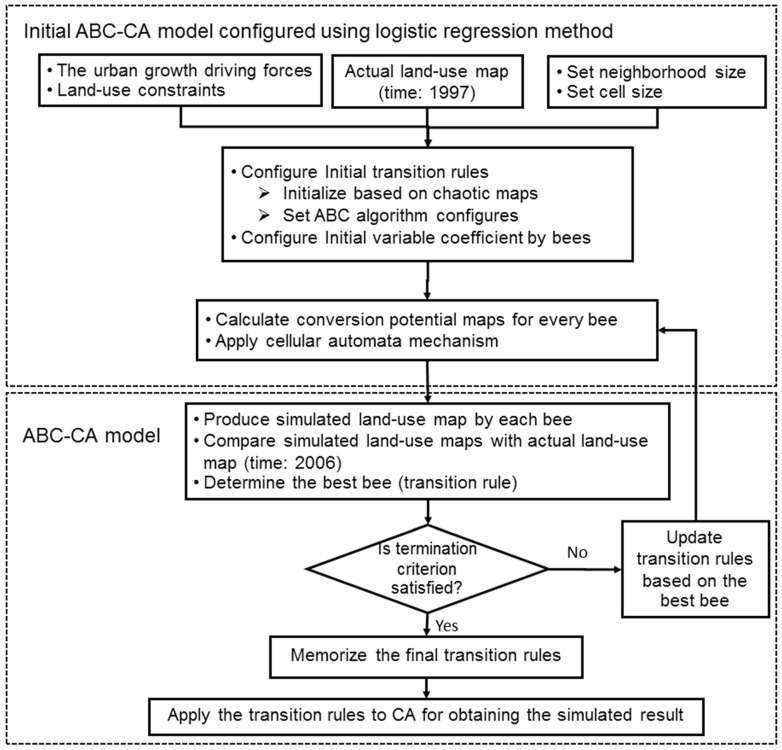
The new ABC-CA urbanization model.

In the proposed model, the number of cells that were not predicted correctly is considered as a cost value ($f_{i}$) of the produced result. This value can be obtained by different cells between simulated and reference maps. Therefore, the fitness function of the proposed model attempts to minimize the cost value. This was repeated in each iteration of the model. The final transition rules provide the estimate of an urban transition for that cell. These rules were applied on the second land-use map by the CA procedure to achieve the predicted map. The pseudo-code of the proposed ABC-CA model is given in Algorithm 2 and Figure 2 shows the flowchart of the new ABC-CA model.
**Algorithm 2**. The pseudo-code of the proposed ABC-CA modelInput datasets Initialize the ABC algorithm configurations Set *Cycle* = 1 **Repeat** until *Cycle* ≤ the maximum iteration number Initialize transition rules using worker bees based on lower and upper threshold values for every variable Calculate conversion potential value (cell status) based on LR and transition rules in CA mechanism  Produce new lower and upper threshold values for new rule construction by the worker bees using Equation (7) Apply the greedy selection process to select worker bees for achieving new lower and upper threshold values Calculate the probability values $p_{i}$ using Equations (8) and (9) **If**
$p_{i}$ > *rand(0,1)*, **then**  Produce new lower and upper threshold values for onlooker bees using Equation (7)  Apply the greedy selection process to select onlooker bees for achieving new lower and upper threshold values **End if** Determine the lower and upper threshold values, if they exist Replace it with a new randomly produced position (the lower and upper threshold values) for the scout using Equation (7) Record current best (the lower and upper threshold values) and construct current best rule Calculate conversion potential value based on LR and transition rules in CA mechanism Produce the simulated land use map and Compare it with actual land use map *Cycle* = *Cycle* + 1 **End repeat**

### Model Assessment

The accuracy of the simulation results compared to the reference map needs to be validated to quantify the goodness-of-fit of urban growth projections [74]. Comparisons between the observed and the simulated changes, based on the four generalization types of pixels, are performed. These pixel types include the areas of observed change simulated correctly (hits; *H*), observed persistence simulated as change (false alarms; *F*), observed change simulated as persistence (misses; *M*), and observed persistence simulated as persistence (correct rejections; *CR*) [52]. Table 2 shows the contingency table representing the proportion of pixels in the actual map versus the simulated maps illustrating the allocation disagreement between the simulated and actual changes. Indeed, the contingency table presents accuracy statistics, including total accuracy, user accuracy and producer accuracy.

Other statistical measures, such as figure of merit (FoM) and overall accuracy (OA), were used to assess the accuracy of the models. The FoM is defined as the ratio of the intersection of observed change and predicted change to a union of the observed change and predicted changes. Also, the OA is calculated by dividing the total number of correct pixels (diagonal) by the total number of pixels in the contingency table. It measures the overall proportion of correctly categorized pixels as changes to the total number of cells [75,76].

Another method that is widely used to validate urban growth and land change models is the receiver operating characteristics (ROC) index that evaluates the capability of the model in producing the best suitability maps independently of applying threshold values [77,78]. However, several researchers have argued against the use of ROC as a model goodness of fit as it has biased outcomes. To overcome ROC deficiencies, the total operating characteristics (TOC) index was introduced [75]. TOC generates a graphical plot to reveal the information which shows the entire contingency table for all thresholds while ROC fails to do that. TOC shows the produced information of ROC and additional information such as the size of the number of observations (as the horizontal axis) and the size of references (the vertical axis). For the TOC plot, there are two minimum and maximum boundaries that show the possible space of appearing the TOC curve. If the curve is close to the maximum boundary, then the ranking of observations is high. Each point with the horizontal value and vertical value in TOC is calculated by Equation (10) and a corresponding threshold *t* [75]:

(10) $$X_{t}=\frac{F}{F+CR}\;;\;Y_{t}=\frac{H}{H+M}$$

In this research, the mentioned validation methods (comparisons between the observed and the simulated changes, OA, FoM and TOC plot) were used to evaluate performance of the implemented methods.

## 5. Study Area and Dataset

**Figure 3:**
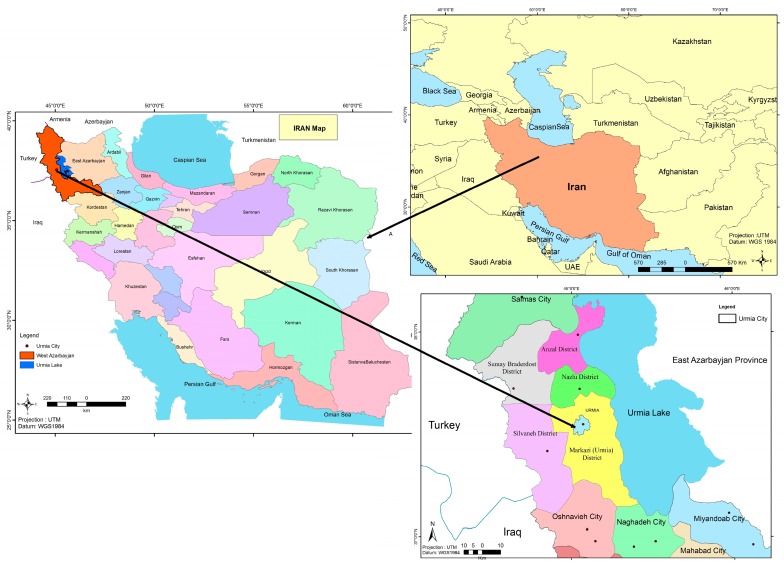
Iran, west Azarbayjan Province and the city of Urmia.

Our new ABC-CA model is tested using spatial data from the city of Urmia, Iran (Figure 3) with the objective of being able to predict its future urban growth patterns. In the past five decades, the population of Urmia has increased 10-fold [69]. However, its area has increased approximately 27-fold during this same period. This means that the rapid urbanization has occurred in this period at an unplanned trend. 

Additionally, a simple land-use change analysis reveals that the areas in northeast and east of the border of the city were agricultural lands in 1997 and were converted to urban areas in recent years. Based on the land use maps, urban lands in Urmia in 1997 were about 4435 hectares, and these lands increased to 7140 hectares in 2006 and 9500 hectares in 2015. This means that the area of Urmia increased by more than double in the recent 20 years. During 1997–2015, about 54% of the urban areas of Urmia were mostly agricultural lands located on the margins of the city. There is local concern that if these trends continue, the agricultural economy of the region will be negatively impacted. To avoid this outcome, this study was initiated in order to forecast the future growth trends of Urmia which can be useful for municipal land use planners.

To develop our ABC-CA urban model, we used as the basic data land use maps that were extracted from a classification of Landsat satellite images of the years 1997, 2006 and 2015 (Landsat 5, 7 and 8, respectively) for the Urmia metropolitan region. We employed a supervised maximum likelihood classification method and registered the land use/cover maps to the National Cartographic Centre of Iran topographic maps at a scale of 1:2000. We classified the Landsat images as three classes including urban areas, agricultural lands and wastelands. The overall accuracies for the classified maps for the years 1997, 2006 and 2015 were 91.73%, 93.38% and 93.55%, respectively.

**Figure 4:**
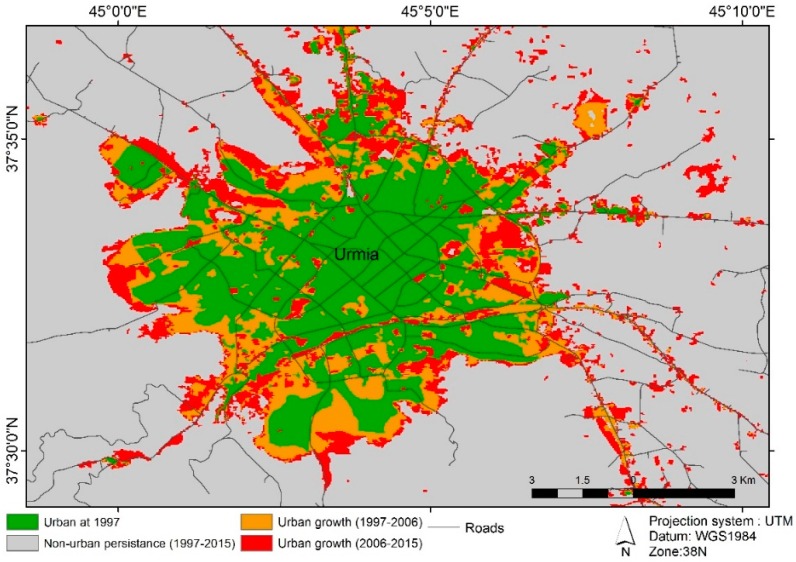
Urmia city urban growth from 1997 to 2015.

Based on these land use maps of the city of Urmia, urban maps for 1997 to 2015 were extracted (Figure 4). According to previous studies of urban growth (e.g., [36,61,79]) the urban growth driving forces used to predict future urban growth; the relevant data are shown in Table 3. The slope and elevation maps with resolution of 30 × 30 m are derived from the digital elevation model extracted from topographic maps produced by National Cartographic Center of Iran.

**Figure 5:**
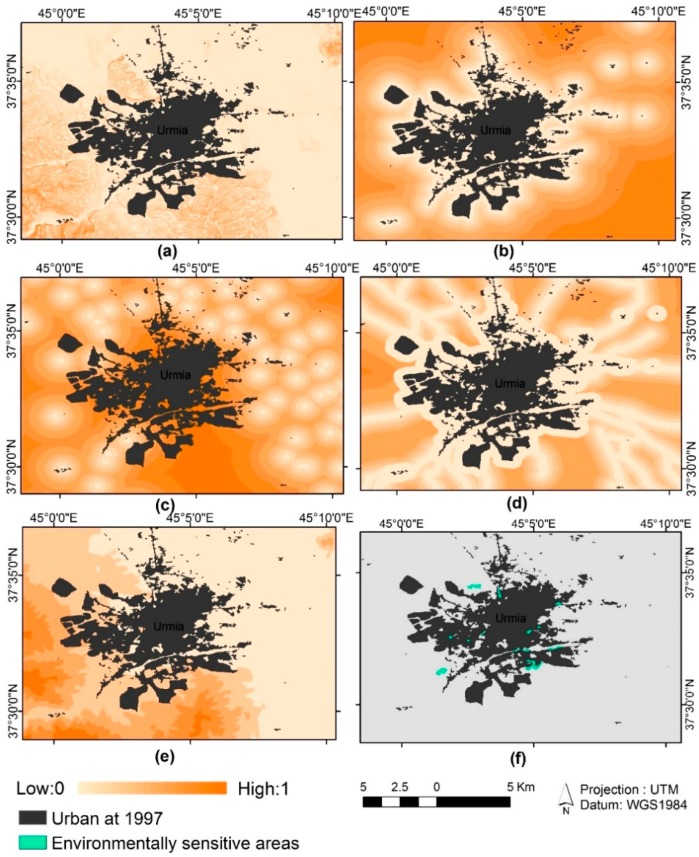
Normalized data of Urmia urban growth process. (**a**) Slope map; (**b**) Proximity to major business centre map; (**c**) Proximity to population centre map; (**d**) Proximity to major roads map; (**e**) Elevation map; and (**f**) Environmentally sensitive areas map.

The slope and elevation maps are important factors in modeling of urban growth because they involve realism into the prediction model by representation of real morphology of the study area. Another dataset used in this study is transportation network that is demonstrated the density of transport network showing the relationships among different land-uses. The road dataset was extracted from the topographic map and updated by the data produced by Road Maintenance and Transpiration Organization of Iran. Another dataset used in this model is environmentally sensitive areas such as forests, wetlands, floodplains, or environmentally sensitive lands that the future urban growth plan must preserve. This data was derived from environmental maps and land use maps. Proximity to major business center of Urmia and its neighboring population centers maps are other datasets used in the study. These datasets can have effect on the city’s future growth. Our investigation shows that in last decades the fast urban growth in Urmia has caused the neighboring villages to join to the city, so the associated data given in Table 3 were collected from different sources, converted to raster format at a spatial resolution of 30 m and loaded to the GIS database in this research (Figure 5).

## 6. Implementation and Results

**Figure 6:**
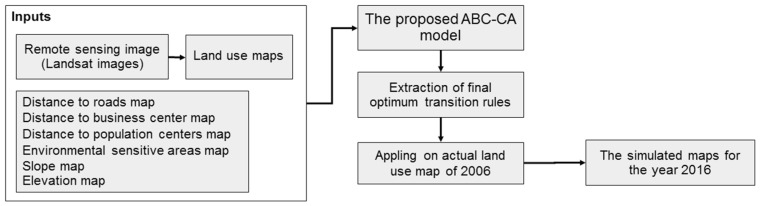
The steps to modeling using our ABC-CA urban model.

The proposed ABC-CA model was developed in Matlab™ and ArcGIS™ software based on the data processing steps presented in Figure 6. Based on the obtained rules applied on the actual land-use of 1997, the simulated map was produced for 2006. To train the model, comparisons of the simulated and actual land-use map of 2006 was made as measures of their goodness of fit, and if their similarities met the threshold criteria, the process was stopped and the algorithm presented the optimum transition rules for CA model; otherwise, it was repeated until reaching the threshold criteria (in this research, the algorithm was repeated 350 times). As it was mentioned earlier, the threshold value of CA part of the model (*Q* in Equation (5)) can be found out through a couple of trials. In the proposed model, the cost value was defined with difference between the simulated and the reference maps. In the iterative approach, the model attempts to minimize the cost value. The land use in 2006 is then simulated by running the ABC–CA model.

In the next step, the obtained optimum transition rules are applied to actual land-use map of 2006 to achieve the predicted map of urban growth of the city of Urmia for 2016. Additionally, to test the model prediction capability, the predicted map is verified by the reference map of 2015. If the verification is accepted, the final optimum transition rules are obtained. Otherwise, some modifications on the ABC algorithm are performed and then the above process (re-training the model to achieve new optimum rules) is repeated until the result is satisfactory through the verification step. Based on trial and error, our model could obtain the final optimum rules with modification of the ABC-CA specifications with ten repeats. This means that the algorithm was repeated 350 times in any iteration of the test step. The evaluation step was performed based on getting to maximum likelihood or minimum difference between the simulated results and the actual land use map.

**Figure 7:**
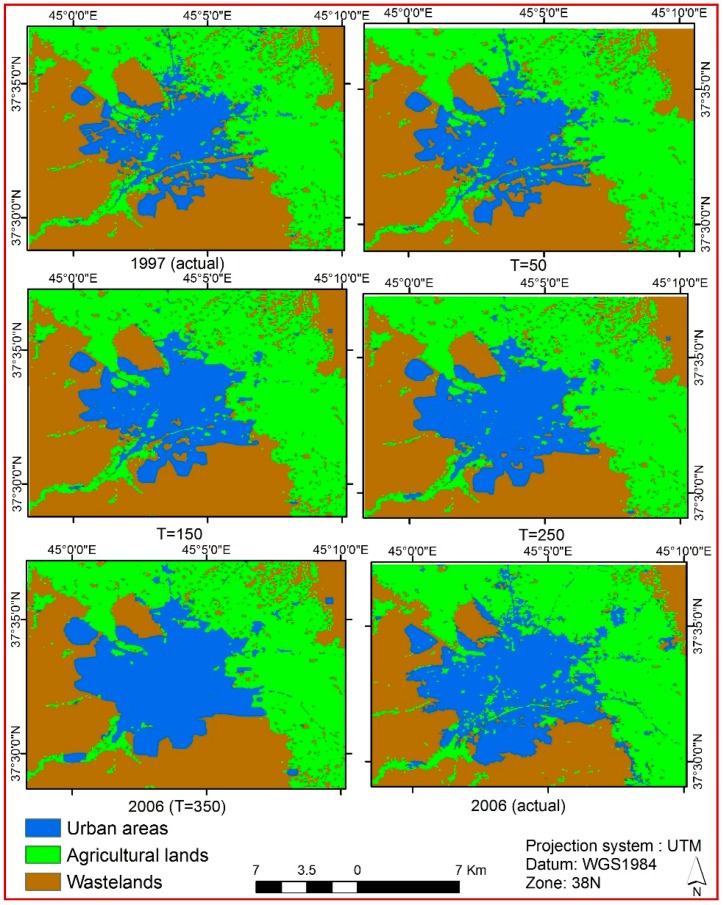
The ABC-CA model simulation results.

Figure 7 illustrates the ABC-CA model results in various iterations, where T is the number of iterations. According to Figure 7, the ABC-CA model had been slow in the simulation process up to iteration 150. This means that the discovered transition rules were not optimal to predict the 2006 land use correctly. However, the model performed with faster trends, especially after iterations 250 through 318. In other words, the model continued to produce more optimized rules between iterations 250 to 318. Since ABC is a stochastic search algorithm, there is no guarantee the results will improve by increasing the number of iterations in the ABC-CA model. Investigation of the corresponding results in various runs of our model show that increasing the number of runs may not guarantee achieving better rules and consequently better simulation and the model should be replicated as long as it is representing a stable outcome. For example, the ABC-CA model operated to improve results up to iteration 13 (extraction of better rules and subsequently producing better simulation results) while the produced result in iteration 14 was worse than the previous iteration and this trend continued to iteration 16. This means that the model could not discover better transition rules and performed worse. However, the model stores the best transition rule and when the result is worse in the next iteration, it discards inappropriate results and continues with new parameters. Subsequently, the model routine is repeated until the results stabilize. Thus, after producing poor results in iterations 14 and 15, our model improved the results and produced better outcomes in iteration 16 compared with iteration 15. It should be noted that more poor results were repeated several times with the ABC-CA model execution during its 350 iterations. However, the behavior of the model was improving the produced results (or transition rules) in ascending order from iteration 248 to iteration 318. Then, our model produced the same results from 318 to 350 iterations (i.e., it was stable).

### 6.1. Model Validation

The proposed model to simulate the growth of Urmia for the year 2006 is run and the model results are produced. To compare the results in operation, the implementation of other SI-based models such as ACO can help to provide a proper understanding of our model’s performance. Several studies have been performed using ACO to discover transition rules of CA in the simulation of urban systems [59,60]. They revealed that ACO is able to discover CA transition rules better than conventional methods. Therefore, in this research the ACO-CA model was implemented to simulate urban growth and its result was compared with our proposed ABC-CA model. We used actual land use of 2006 as the training data in setting up the model suggested by Pontius and Malanson [74]. For model accuracy assessment, they suggested using the entire landscape. Furthermore, actual land use in 2015 was used to validate the models’ capabilities in producing simulation results.

**Figure 8:**
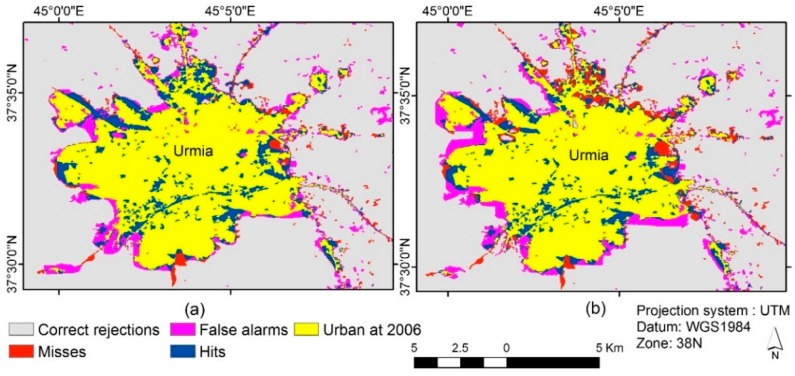
Urban growth simulation models results for the year 2015 with their correctness and errors. (**a**) ABC-CA model; and (**b**) ACO-CA model.

Table 4 shows the two models’ contingency tables representing the proportion of pixels in the actual map of 2015 versus the simulated maps of the two models illustrating the allocation disagreement between the simulated and actual changes. Visual comparison of Figure 8 indicates that distributions of allocation errors (misses and false alarms) in the ABC-CA model result are fewer than the ACO-CA model result, while the ABC-CA model has slightly more hits. In other words, the spatial patterns of the simulated urban growth have more compliance with actual urban growth trends compared to the other method. These findings are also confirmed by Table 4.

In this research, the FoMs of the ABC-CA and the ACO-CA models were 51.7% and 42.9%, respectively. This means that the FoM of the proposed models had better agreement between the observed and predicted changes. Also, the OA is calculated by dividing the total number of correct pixels (diagonal) by the total number of pixels in the contingency table [77]. The OA of the ABC-CA and the ACO-CA models were 90.1% and 87.2%, respectively. This means that the ABC-CA model could produce better prediction accuracy. The allocation disagreement (AD), summation of the false alarms and misses, is another index for validation of the model results. The AD of the ABC-CA model was 9.9%, while this measurement for the ACO-CA model was 12.8%. The amount of disagreement that occurred due to the ABC-CA model simulation is less than with the ACO-CA model. The results also show that the ABC-CA model outperforms the ACO-CA model with fewer quantity and allocation errors and slightly more hits.

**Figure 9:**
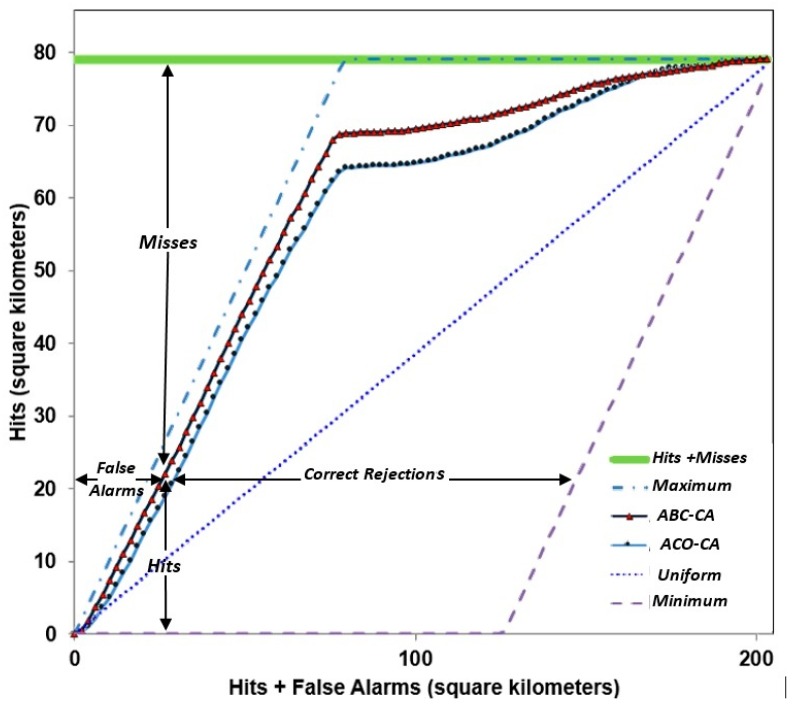
The TOC plot of the ABC-CA and ACO-CA models.

Figure 9 shows the TOC plots for the ABC-CA and the ACO-CA models. Comparing the two plots shows that the TOC curve of the ABC-CA model is closer to the maximum boundary representing the highest ranking observations of the index variable [75] rather than the TOC curve of the ACO-CA model. This means that the ABC-CA model produces better results compared to those of the ACO-CA model.

### 6.2. Results and Discussion

**Figure 10:**
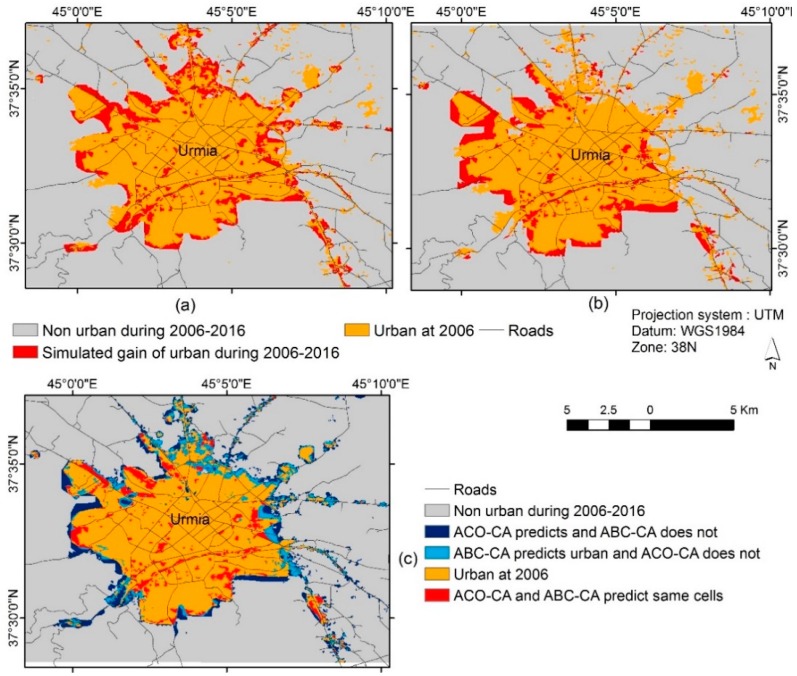
Urmia city prediction results for 2016. (**a**) ABC-CA model; (**b**) ACO-CA model; and (**c**) ACO-CA and ABC-CA models comparison.

After the validation process, the new ABC-CA model was applied to predict the future of urban growth in Urmia for the year 2016 (Figure 10).

Therefore, comparing the boundary of Urmia in 2006 and its prediction for 2016 illustrates that urban growth in Urmia will most often occur in the northwest, west and southern areas of the city that are mostly barren lands. These sites have suitable specifications to undergo urban development showing the planned urban growth. Figure 10 shows the cells where ACO and ABC models agree and disagree for the predictions. According to Figure 10, in 34.1% of the growth cells, the ABC-CA and the ACO-CA models predict similar results.

Furthermore, based on Figure 2 urban growth in Urmia during 2006–2015 is 2326 hectares (25,839 pixels) that the ABC-CA model could simulate 63.3% of the growth correctly while the ACO-CA model predicted 45.6% correctly. In other words, the cells where the ABC-CA predicts as urban cells correctly are about 18% more than the ACO-CA predicts as urban. In this study, swarm intelligence (SI) algorithms, in particular the ABC algorithm, was integrated with CA to calibrate CA in the urban growth process because of its capabilities in dealing with complex relationships. Based on the spatial and temporal properties of the proposed model, modelers can combine complex tools to distinguish long-term trends in urban growth from local changes. The simulations presented here verify that the city tends to expand in bordering areas except in agricultural areas in the northeast. According to the modeling results, it was determined that most of the urban growths in Urmia in 1997–2015 occurred in the boundaries of the city with the conversion of rural land to urban regions. The proposed model results follow up the existing urban growth trends partially, but the model tried to produce the results that met the conditions based on urban growth effective parameters.

Due to the nonlinearity and heterogeneity existing among urban growth driving forces, our proposed model could predict the urban growth in Urmia more accurately. The results verified that the model had some errors in predicting changes in the eastern parts of Urmia because the land use of those regions during 1997–2006 remained as agricultural land which were suddenly converted to urban lands in 2015. Our studies indicated that the conversion of agricultural lands to urban areas in the eastern parts of Urmia occurred because of their proximity to a major ring road (connecting the eastern parts of Urmia to its northern parts). It was also found that proximity to roads compared with other distance-based parameters in urban growth in Urmia has played a prominent role in this process. Overall, it was realized that urbanization in the eastern and northern parts of Urmia (especially in northeastern parts) rather than other regions was caused because of the conversion of agricultural lands to urban areas, while in other areas urban growth occurred in the steep fringe wastelands. Meanwhile in this study, the importance of slope and elevation as urban driving forces were assessed lower than other parameters.

However, in this AI hybrid modeling approach, a number of limitations were encountered. The model produced false growth values or missed the growths so errors were possible. In some cases, modeling errors arise from errors in the data such as the classification errors in producing land use maps. These errors affected the simulation results shown as the areas in red color (misses) in the southeast and northeast of Urmia. The model cannot provide proper simulation results when there is no prior evidence of the region to growth. In addition, dynamicity of urban growth driving parameters during the simulated period may have effect on spatial distribution of the simulation results.

In terms of performance, the convergence in the ABC-CA model was much faster compared to that of the ACO-CA model because of large search space in urban growth modeling. In addition, implementation of the ABC-CA model was easier than the ACO-CA model because the ABC algorithm is considered highly flexible since only requires two control parameters of maximum cycle number and colony size, while the ACO algorithm has more control parameters. The ACO algorithm was usually used to solve discrete problems such as network-based problems (finding a shortest path) and it needs discretized attributes but the ABC algorithm can be used flexibly in continuous (urban growth modeling) or discrete fields.

In this experiment, the performance of the two models for modeling urban growth in Urmia are assessed using Matlab2010 on a CORE i7 CPU with 8GB RAM. The time taken (in seconds) to complete achieving the outcomes for the ABC-CA model is 31.6 h, while the ACO-CA model took 39.3 h to complete the process. However, our proposed model, to obtain accurate results, generally performs slowly because of many iterations required. The computational cost of the algorithm is extensive and it requires a high memory capacity. To overcome these limitations, it is necessary to consider some strategies such as using a new search mechanism, improvement in the configuration of the initial population and using the neighborhood selection by onlooker bees [81,82] to improve the algorithm performance.

Finally, in this research we attempted to propose a new urban growth modeling with the CA model that integrated with the ABC algorithm. The OA index of the proposed model compared to the ACO algorithm has not been a significant improvement (<3%), however in all evaluation indices including running time, convergence capability, flexibility, statistical measurements and the produced spatial patterns, the ABC-CA model’s performance showed relative improvement and therefore its superiority was confirmed.

## 7. Conclusions

In CA models, as urban change models, extraction of transition rules which determine the future status of the cells is critical. It is necessary to use proper methods (AI) to discover the rules because of special conditions such as nonlinearity of city expansion. A new method based on SI is ABC. The ABC algorithm performance in solving optimization problems is good. However, few studies have employed the ABC algorithm for urban growth modeling. We constructed an ABC-CA model that has better performance in numerical tests compared with those of the other similar methods. In this study to calibrate the CA model, the integration of LR and the ABC optimization approach has been presented and assessed. We selected 1997 as the base year for simulating future urban growth of Urmia and used land uses of years 2006 and 2015 to test and validate results, respectively. Finally, the simulation results were obtained for the urban growth in 2016. Assessments of the results of the proposed model and ACO-CA model were performed using overall accuracy and error as diagnostic tools, AD, FoM, OA and the TOC curve. The overall accuracy and the figure of merit of the ABC-CA model is 90.1% and 51.7%, which is 2.9% and 8.8% higher than those of the ACO-CA model, respectively. The allocation disagreement of the ABC-CA model is 9.9%, which is 2.9% less than that of the ACO-CA model. The TOC curve of the ABC-CA model is closer to the maximum boundary representing the highest ranking observations of the index variable rather than the TOC curve of ACO-CA model. Therefore, the prediction results for 2016 show that the ACO-CA model has more urban growth rate (15% more) compared with those of the ABC-CA model.

## Figures and Tables

**Table 1:** Part of the transition rules derived by the proposed models.

**If** (Dist-road < 0.400 km & Dist-road > 0.200 km & Slope > 0% & Slope < 3% & Elevation > 1250 m & Elevation < 1320 m & Dist-Business_center < 2 km & Dist-Business_center < 0.5 km & Dist_population_center > 0.5 km & Dist_population_center < 2 km & Neighborhood_Info < 8), **Then** Probability of conversion of the cell base on Equations (3) and (4): (*P* = *0.6755*) **If** *P* > threshold **Then** Pixel class = urban **else** pixel class = non-urban **End if**

**Table 2:** Contingency table.

	Reality			
Model results		**Change**	**Persistence**	**Total (Producer Accuracy)**
	Change	*H*	*F*	*H + F*
	Persistence	*M*	*CR*	*M + CR*
	Total (user accuracy)	*H + M*	*F + CR*	*H + F + M + CR*

**Table 3:** The urban growth driving forces [23,79,80].

Variables	Data Sources
Distance from business center map	Topographic maps (for years of 1997 and 2006)
Distance from road networks map map	Road networks (for year of 1997)
Distance from population centers mapcenters	Topographic map (for year of 1997)
Land use and Land cover maps	Landsat™ classified images (for years of 1997, 2006 and 2015)
Environmental sensitive areas map	Environmental maps (for years of 1997 and 2006)
Slope and elevation maps	Topographic map and digital elevation model (for year of 1997)

**Table 4:** Contingency tables of the ABC-CA and the ACO-CA models.

	Reality (the ABC-CA Model)				Reality (the ACO Model)		
Simulation results		**Change**	**Persistence**	**Total**	**Change**	**Persistence**	**Total**
	Change	10.6%	6.7%	17.3%	9.6%	7.9%	17.5%
	Persistence	3.2%	79.5%	82.7%	4.9%	77.6%	82.5%
	Total	13.8%	86.2%	100%	14.5%	85.5%	100%
